# Supplementary material for: Inhibition of oxygen dimerization by local symmetry tuning in Li-rich layered oxides for improved stability
Source: Nat Commun. 2020 Oct 2;11:4973. doi: 10.1038/s41467-020-18423-7 (PMC7532436; doi:10.1038/s41467-020-18423-7)
Supplement: Supplementary file 1 — Supplementary Information [file 41467_2020_18423_MOESM1_ESM.pdf]

Supplementary Information

Inhibition of oxygen dimerization by local symmetry tuning in Li-rich layered oxides for improved stability

Ning et al.

Supplementary Figures

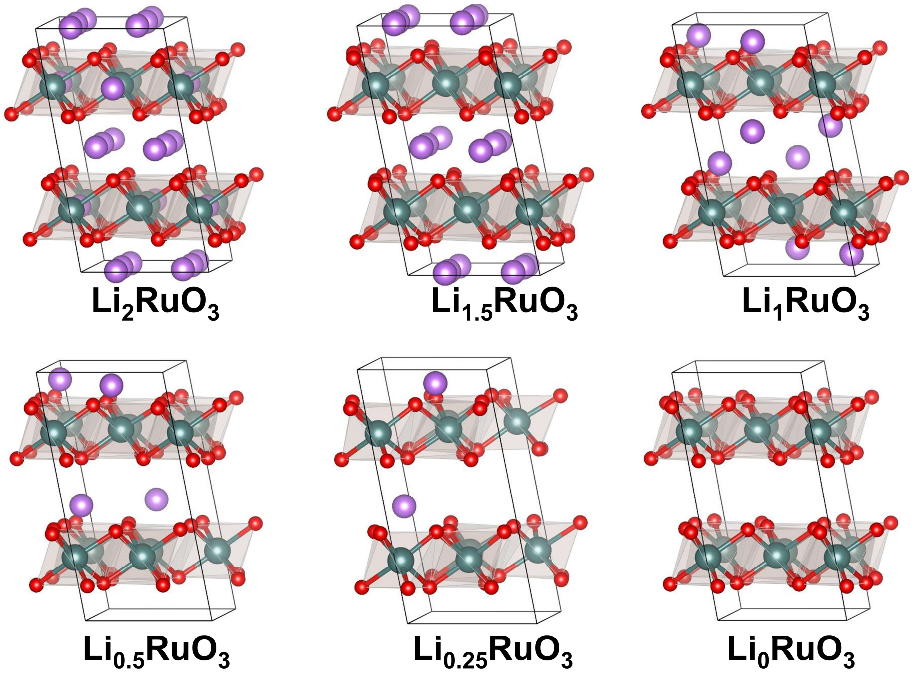

Supplementary Figure 1. The optimized structures upon delithiation. The lowest energy structures of R-$Li_{2-x}RuO_3$ for x = 0, 0.5, 1.0, 1.5, 1.75, 2.0. The purple, dark cyan, and red spheres are Li, Ru, and O, respectively.

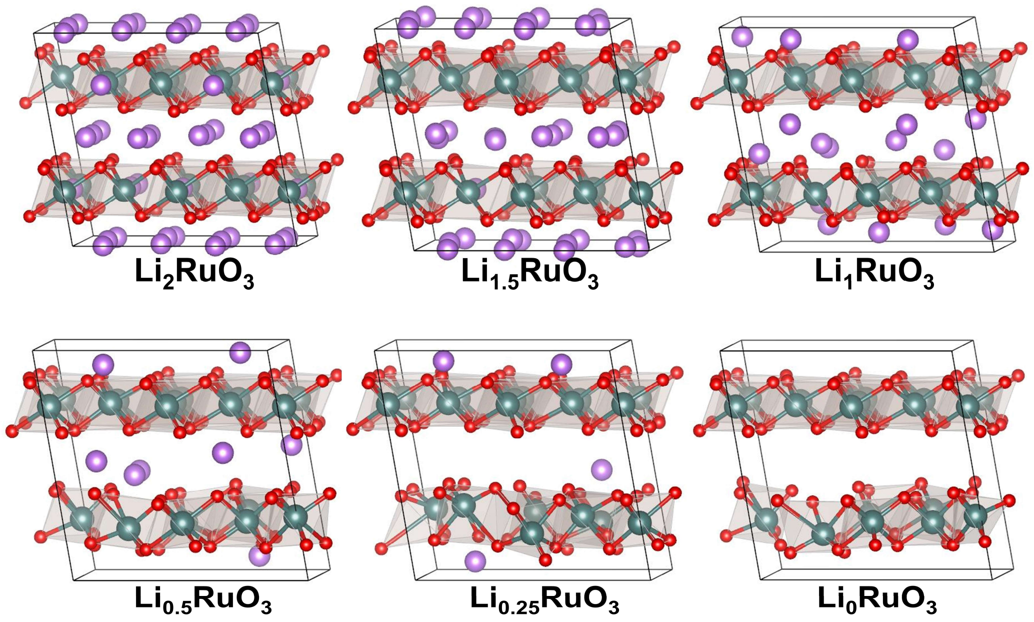

Supplementary Figure 2. The optimized structures upon delithiation. The lowest energy structures of ID-$Li_{2\text{-}x}RuO_3$ for x = 0, 0.5, 1.0, 1.5, 1.75, 2.0. The purple, dark cyan, and red spheres are Li, Ru, and O, respectively.

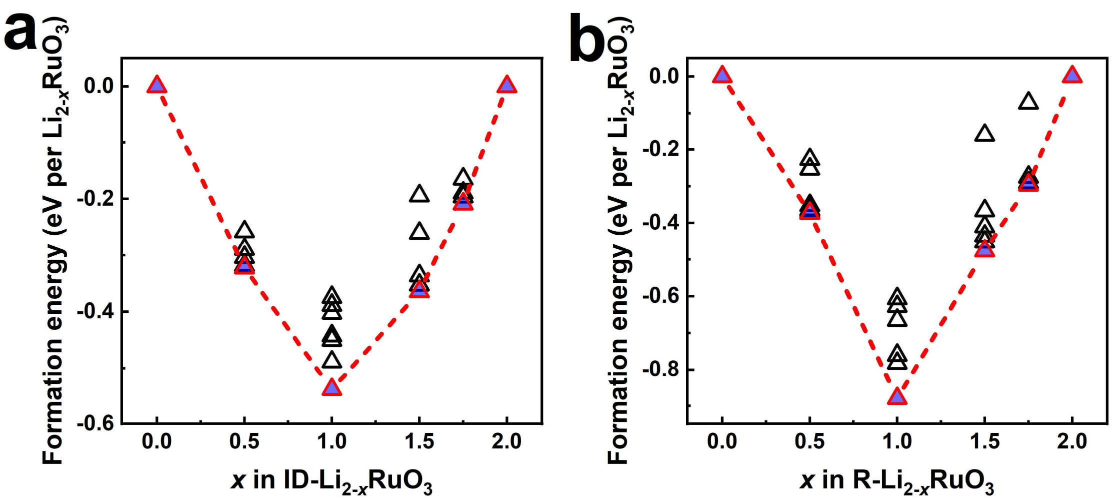

Supplementary Figure 3. The delithiation formation energy. a,b The delithiation formation energy of ID-$Li_{2-x}RuO_3$ (**a**) and R-$Li_{2-x}RuO_3$ (**b**). The formation energy is defined as $E_f(Li_{2-x}RuO_3) = E(Li_{2-x}RuO_3) - 0.5(2-x)\ E(Li_{2-x}RuO_3) - 0.5\ x\ \mathrm{E}(RuO_3)$.

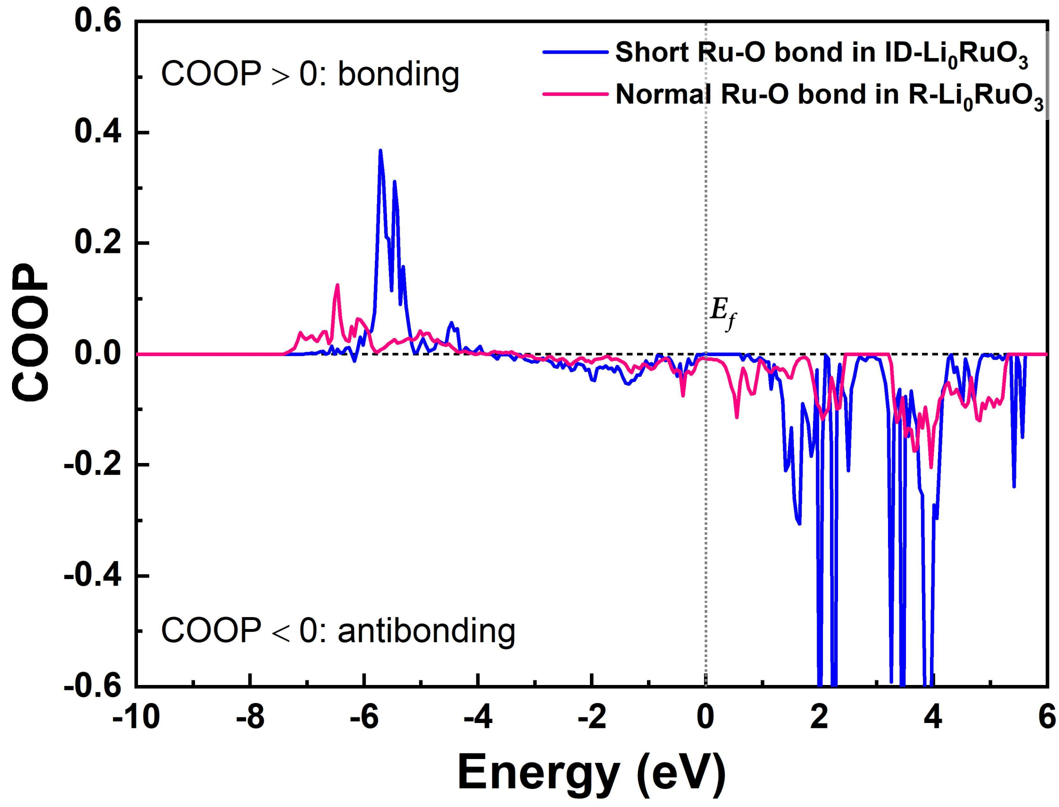

Supplementary Figure 4. The crystal orbital overlap population. The crystal orbital overlap population (COOP) results of the short Ru–O bond in ID-Li_0RuO_3 and normal Ru–O bond in R-Li_0RuO_3.

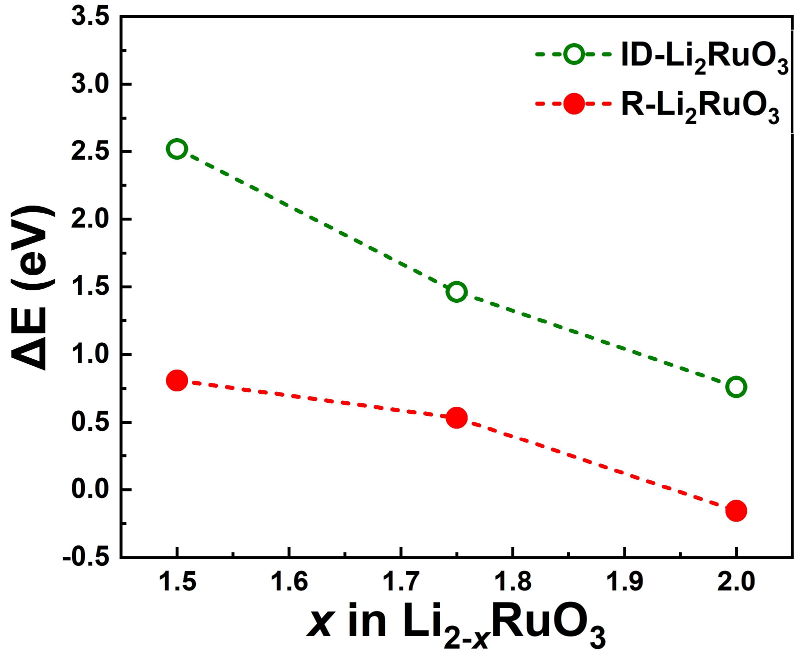

Supplementary Figure 5. The formation energy of Ru anti-site defects. The formation energy (ΔE) of Ru anti-site defects (Ru migrate to octahedral sites of Li layer) in deep delithiated ID-$Li_{2-x}RuO_3$ and R-$Li_{2-x}RuO_3$ (x = 1.5, 1.75, 2). The same Ru anti-site defects concentration of 1/16 (6% of the total Ru content) is considered for both ID-$Li_{2-x}RuO_3$ and R-$Li_{2-x}RuO_3$.

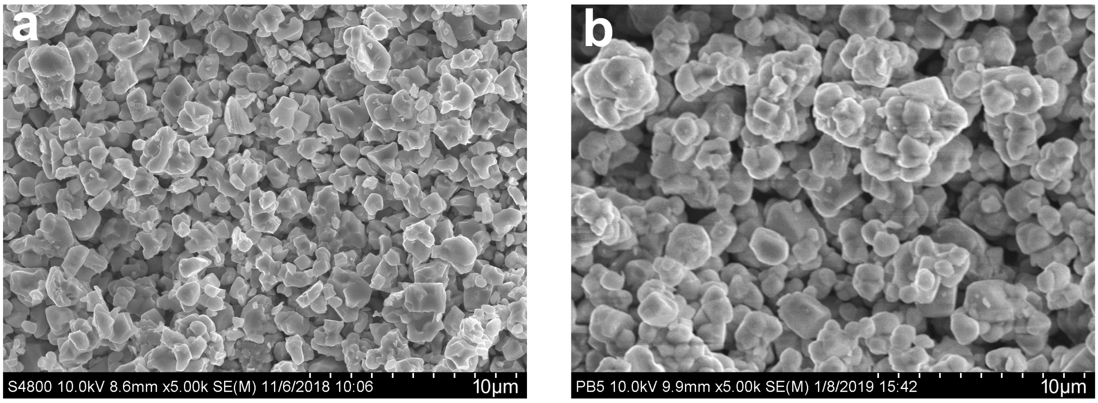

Supplementary Figure 6. SEM images. a,b SEM images of ID-Li_2RuO_3 (**a**) and R-Li_2RuO_3 (**b**).

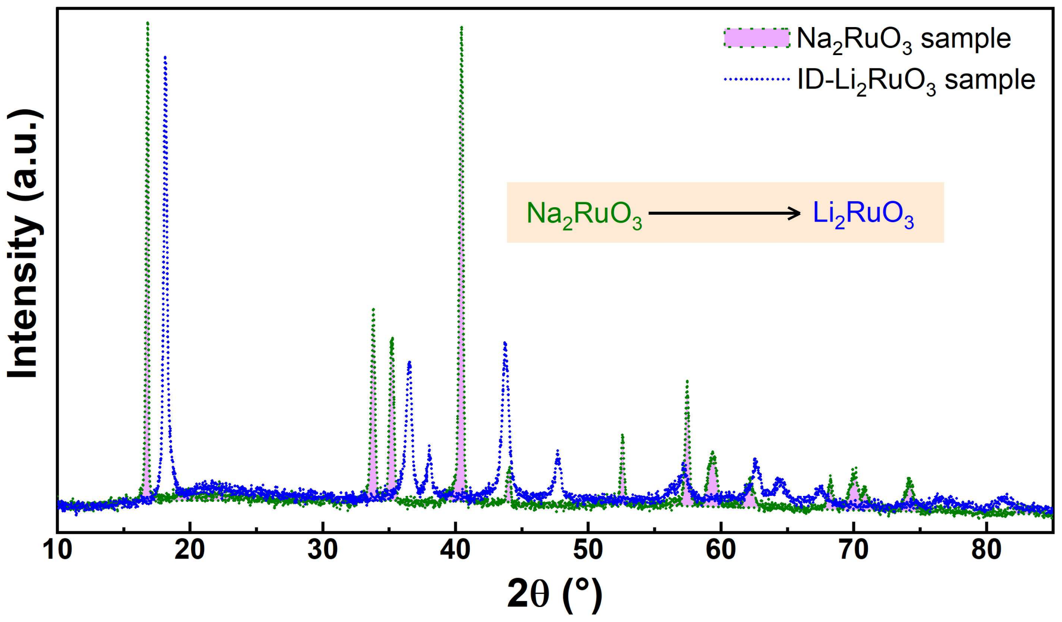

Supplementary Figure 7. XRD patterns of Na_2RuO_3 and ID-Li_2RuO_3. The ID-Li_2RuO_3 sample was obtained by Li/Na-ion exchange of the Na_2RuO_3 sample.

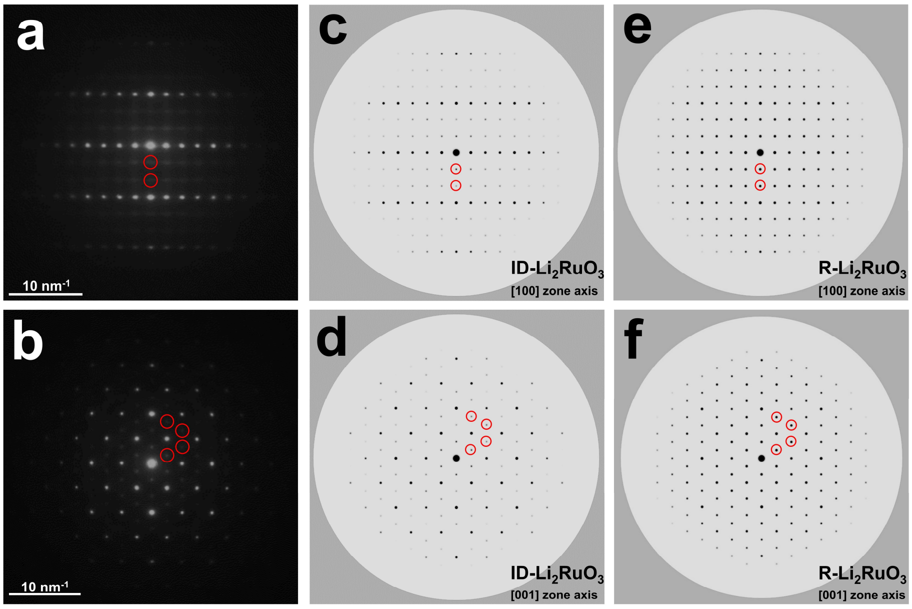

Supplementary Figure 8. The observed and simulated SAED patterns. a,b The observed SAED patterns (**a-b**). The weaker diffraction spots are marked with red cycles. **c-f** The simulated SAED patterns of ID-Li_2RuO_3 and R-Li_2RuO_3 structure models along [100] (**c-d**) and [001] (**e-f**) zone axes, respectively. The ID-Li_2RuO_3 and R-Li_2RuO_3 structure models with C2/m space group used for SAED simulation are taken from the XRD refinements.

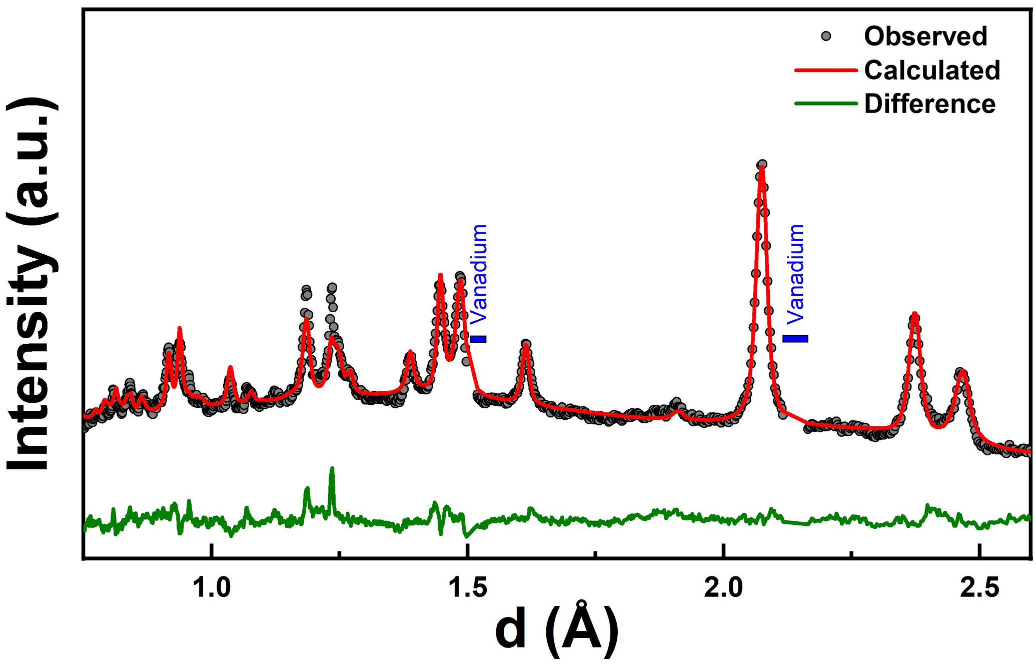

Supplementary Figure 9. NPD patterns of ID-Li_2RuO_3. The impurity peaks of the vanadium pot are omitted within the marked ranges.

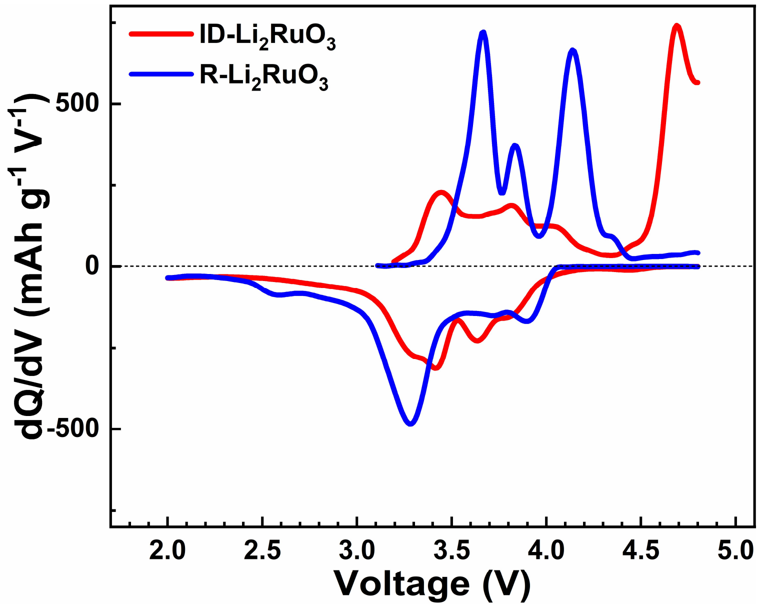

Supplementary Figure 10. The dQ/dV plots. The dQ/dV plots of ID-Li_2RuO_3 and R-Li_2RuO_3 cathodes tested in the voltage range of 2.0–4.8 V at a current density of 30 mA g^{-1}.

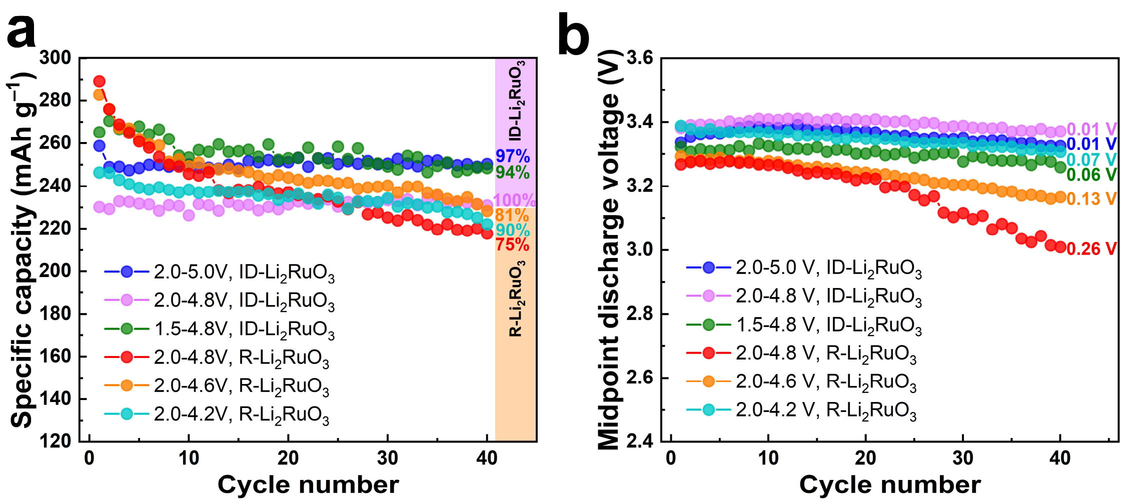

Supplementary Figure 11. Cycling stability. a,b Cycling performance (**a**) and midpoint discharge voltage (**b**) of ID-Li_2RuO_3 in the voltage ranges of 2.0–5.0 V, 2.0–4.8 V, and 1.5–4.8 V compared with those of R-Li_2RuO_3 in the voltage ranges of 2.0–4.6 V, 2.0–4.8 V, and 2.0–4.2 V at a current density of 30 mA g^{-1}.

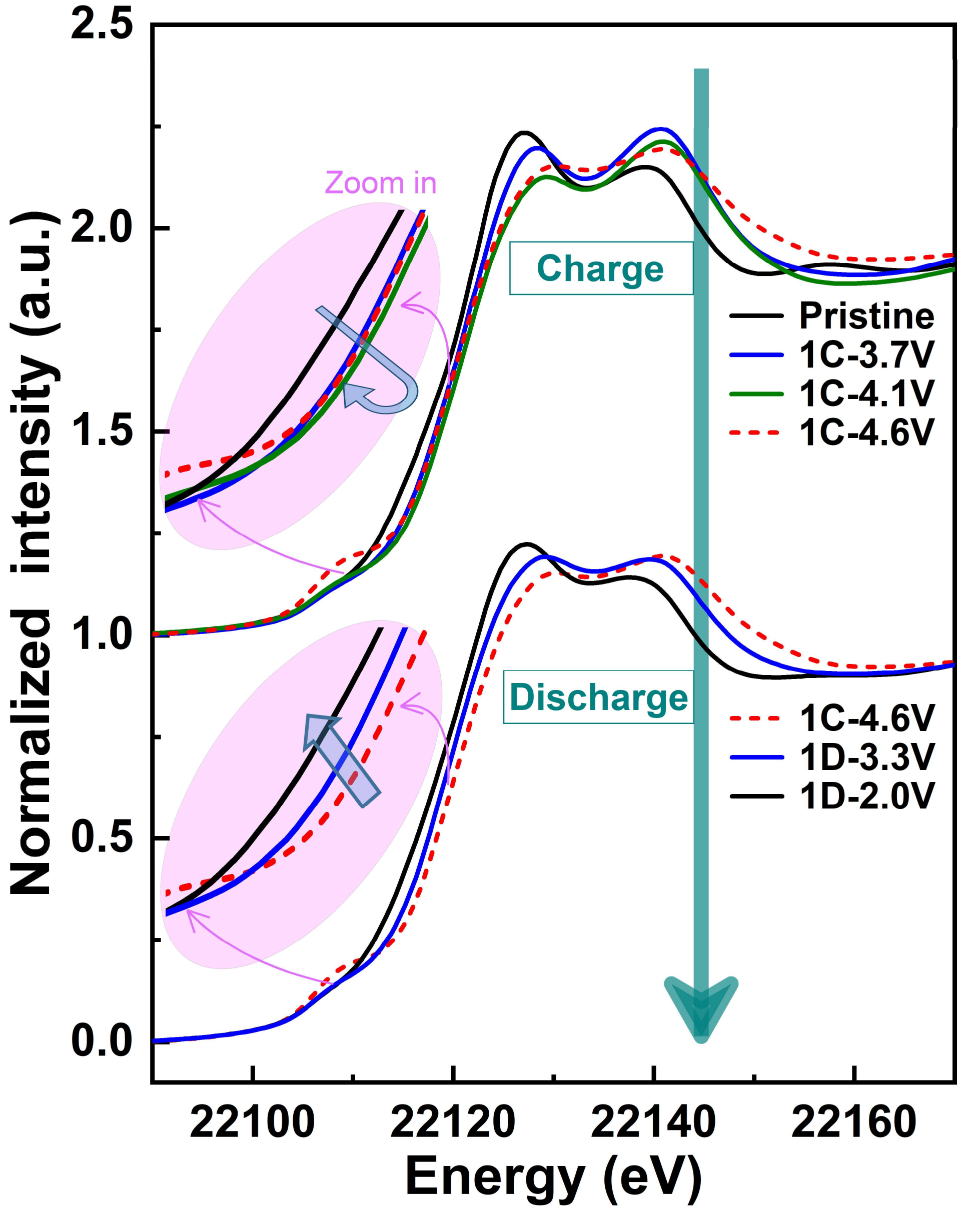

Supplementary Figure 12. Ru K-edge XANES spectra. Ru K-edge XANES spectra of R-Li_2RuO_3 during charge and discharge processes. 1C, 1D represent the first charge and discharge, respectively.

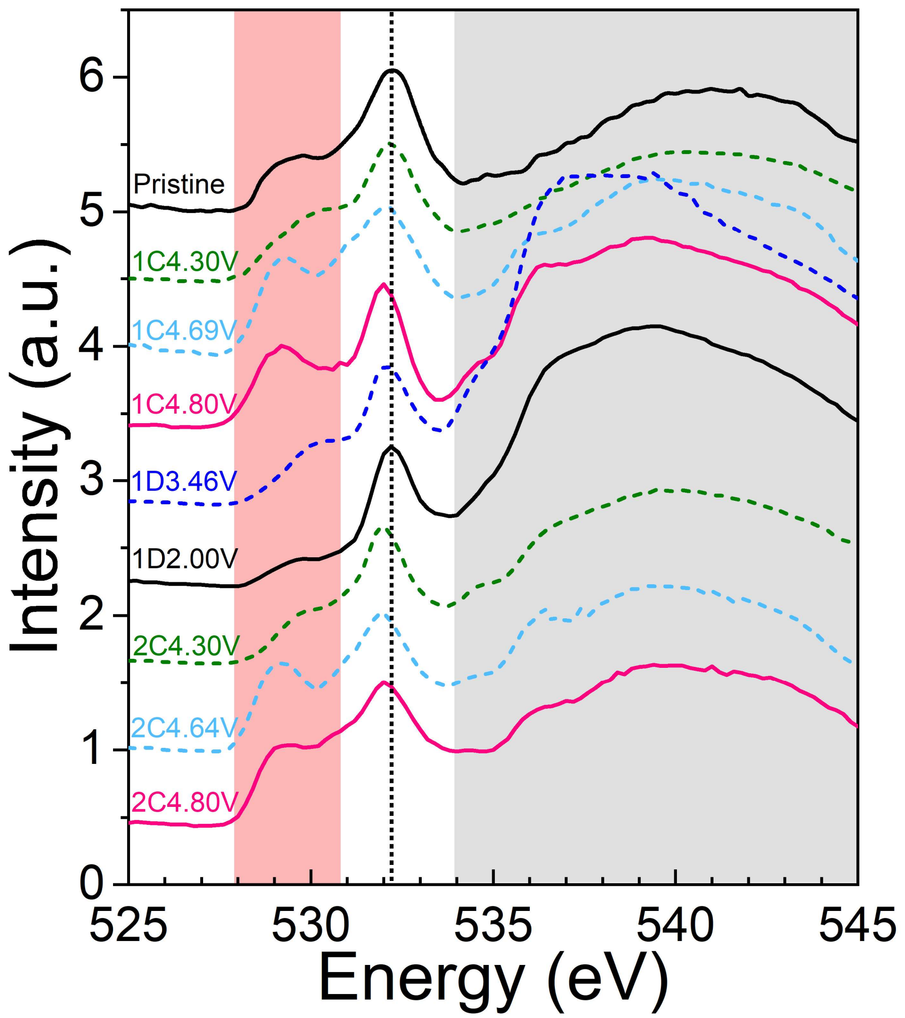

Supplementary Figure 13. O K-edge XANES spectra. *Ex situ* O K-edge XANES spectra of ID-Li_2RuO_3 at various states of charge. 1C, 1D and 2C represent the first charge, first discharge and second charge, respectively.

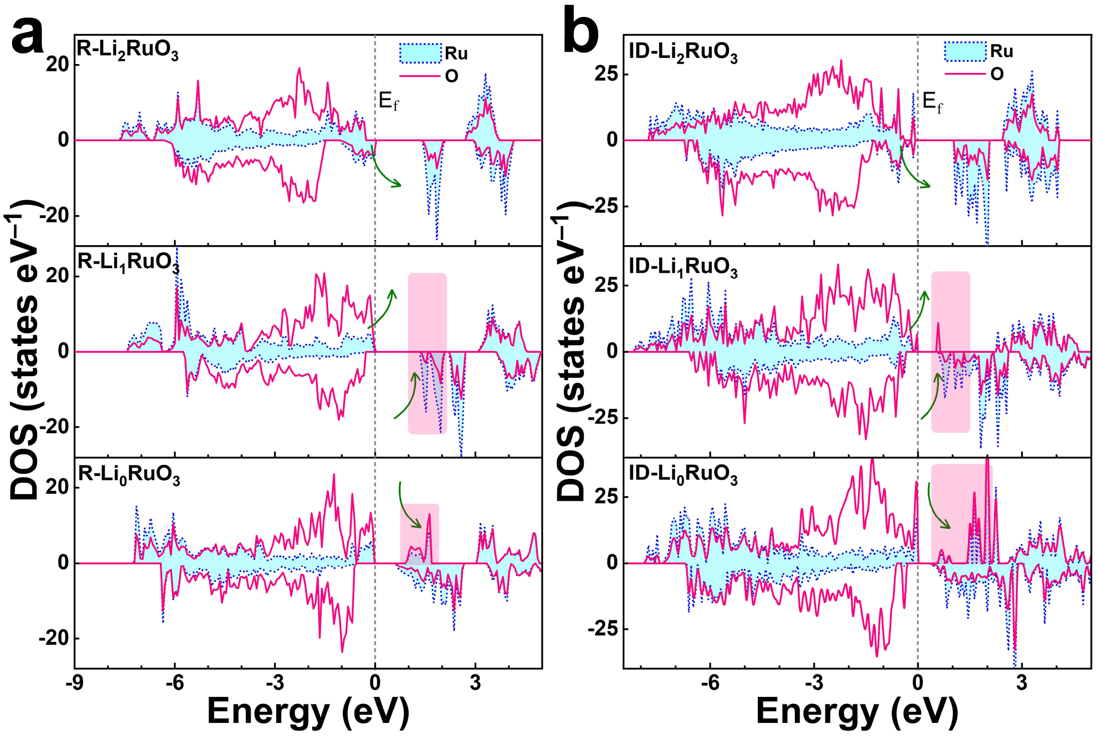

Supplementary Figure 14. Density of states (DOS). a,b DOS of R-$Li_{2-x}RuO_3$ (**a**) and ID-$Li_{2-x}RuO_3$ (**b**) systems with respected to the Li content.

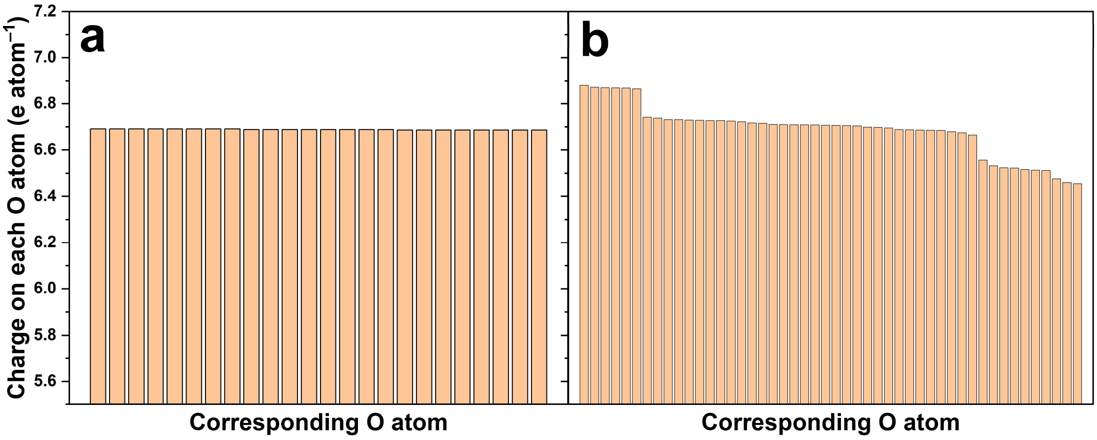

Supplementary Figure 15. Bader charge analysis. a,b Charge on each O ion in fully delithiated R-Li_2RuO_3 (**a**) and ID-Li_2RuO_3 (**b**) systems, as obtained from Bader charge analysis.

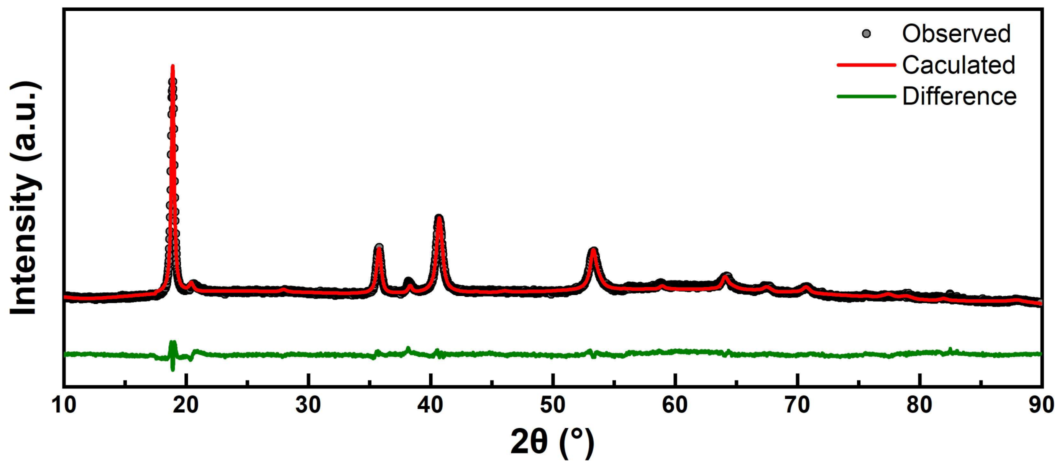

Supplementary Figure 16. The refinement of XRD patterns. The refinement of XRD patterns of ID-Li_2RuO_3 that charged to 4.8 V.

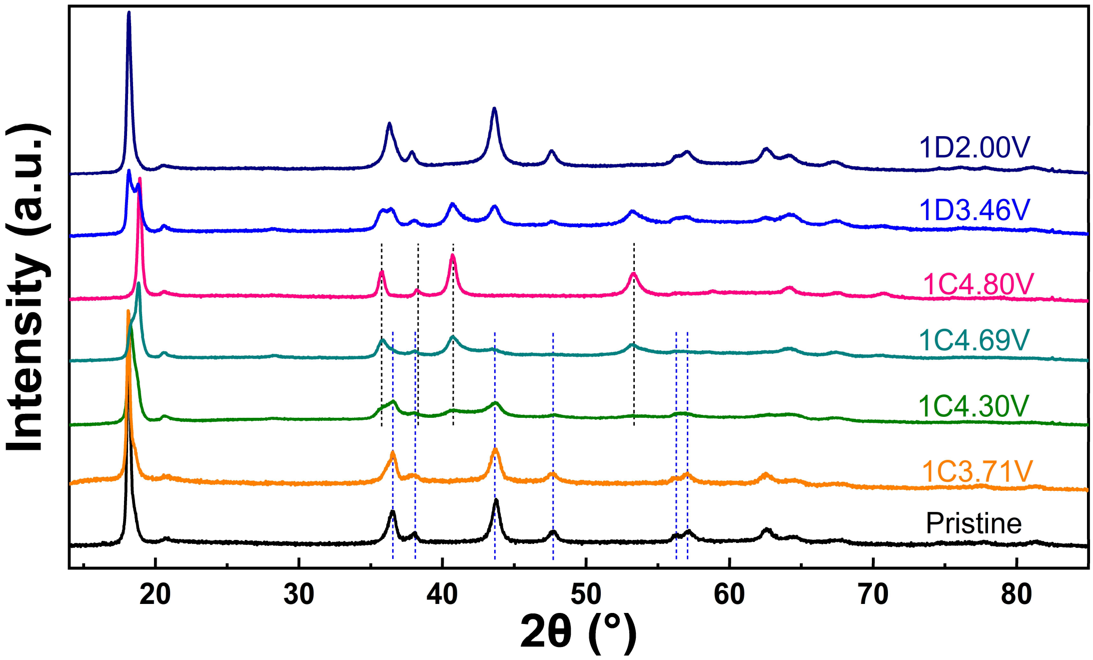

Supplementary Figure 17. XRD patterns of ID-Li_2RuO_3. The *ex situ* XRD patterns of ID-Li_2RuO_3. 1C, 1D represent the first charge and discharge, respectively.

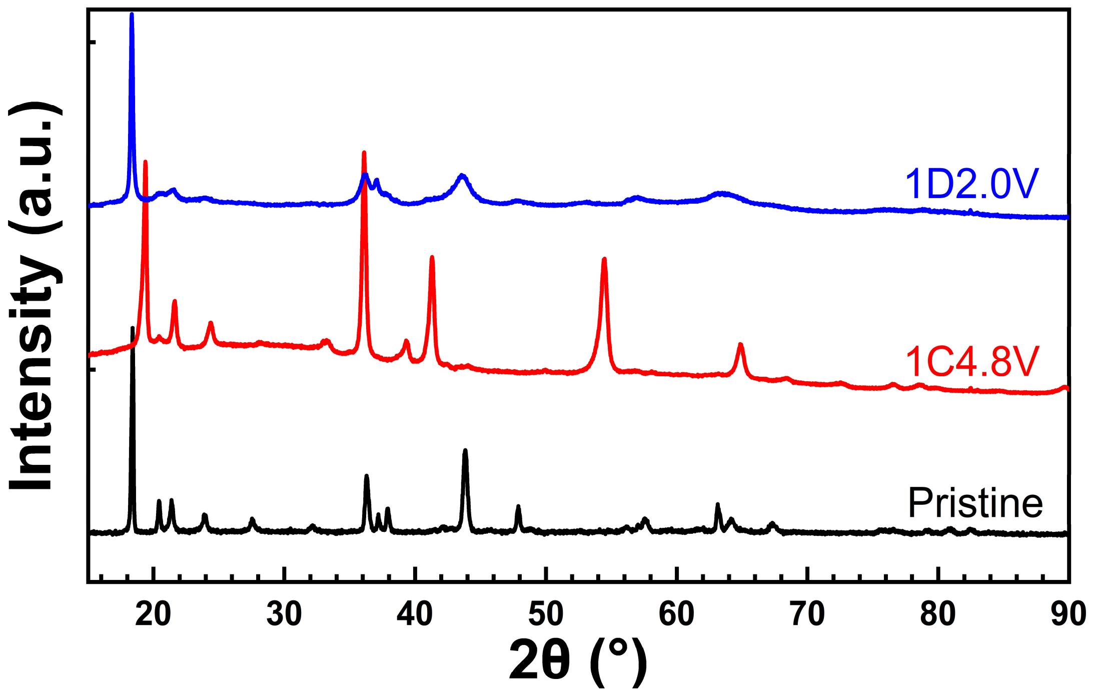

Supplementary Figure 18. XRD patterns of R-Li_2RuO_3. The *ex situ* XRD patterns of R-Li_2RuO_3 during charging and discharging. 1C, 1D represent the first charge and discharge, respectively.

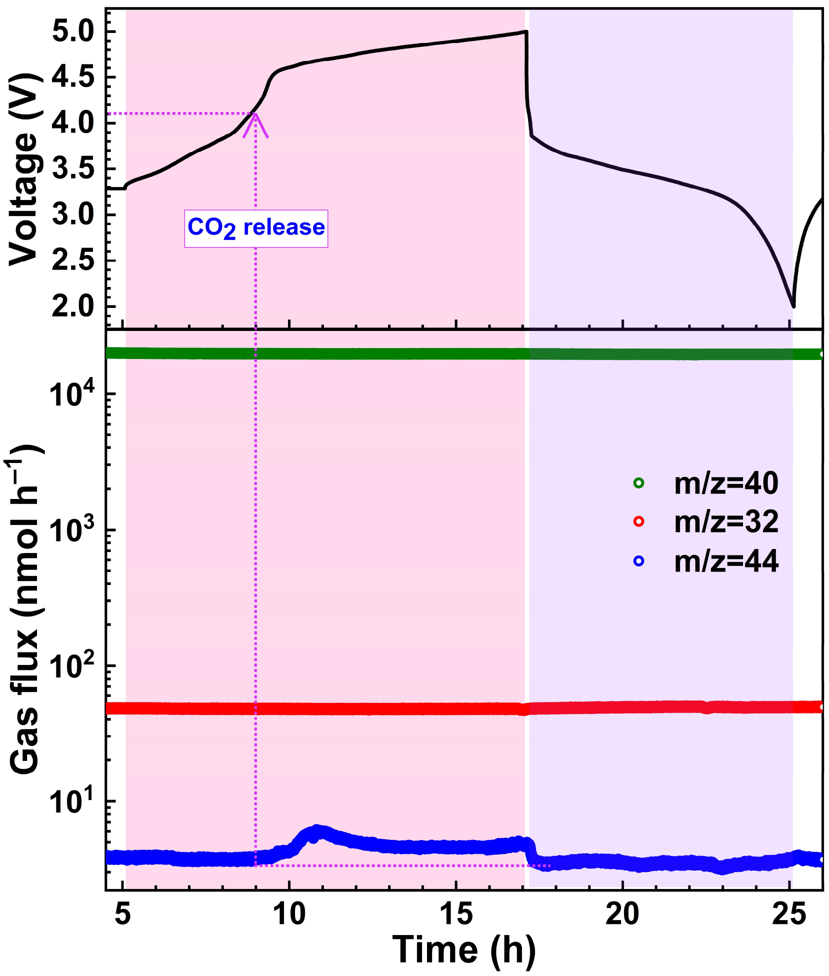

Supplementary Figure 19. Differential electrochemistry mass spectrometry (DEMS). Gas evolution detected by *in situ* DEMS in the ID-Li_2RuO_3 vs. Li cell in the voltage range of 2.0–5.0 V at a current density of 30 mA g^{-1}.

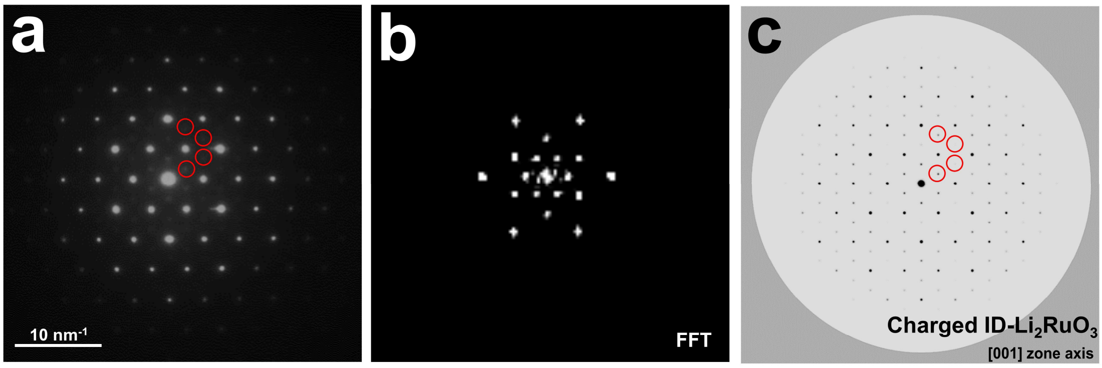

Supplementary Figure 20. Observed and simulated SEAD Patterns. a The observed SEAD patterns of 4.8V charged ID-Li_2RuO_3, **b** the FFT patterns from ABF-STEM image of 4.8V charged ID-Li_2RuO_3, and **c** simulated SEAD of the O1-type structure with Li/Ru disordering arrangement obtained from XRD refinement of 4.8V charged sample.

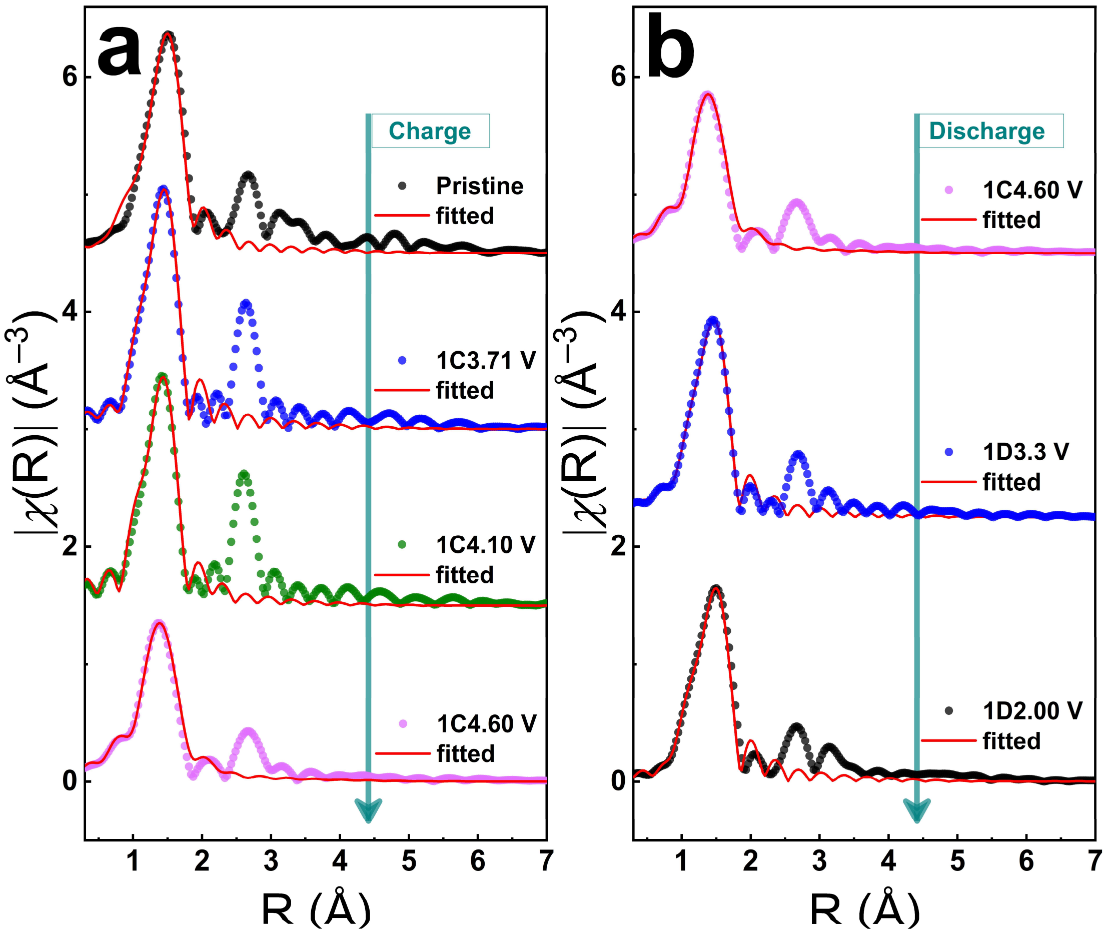

Supplementary Figure 21. The Fourier transform of EXAFS of Ru K-edge. a,b Fitting results for the magnitude of the Fourier transforms performed on k^2-weighted EXAFS oscillations of R-Li_2RuO_3 during the first charge (a) and discharge (b) processes in the R-range of 1–2 Å (first peak only). E_0 was set to −0.7 eV, and the amplitude reduction factor (S_0^2) was fixed at 0.95. 1C, 1D represent the first charge and discharge, respectively. Two group of Ru–O bonds were considered during fitting based on the presence of two crests in the Ru K-edge XANES spectra shown in Supplementary Figure 12.

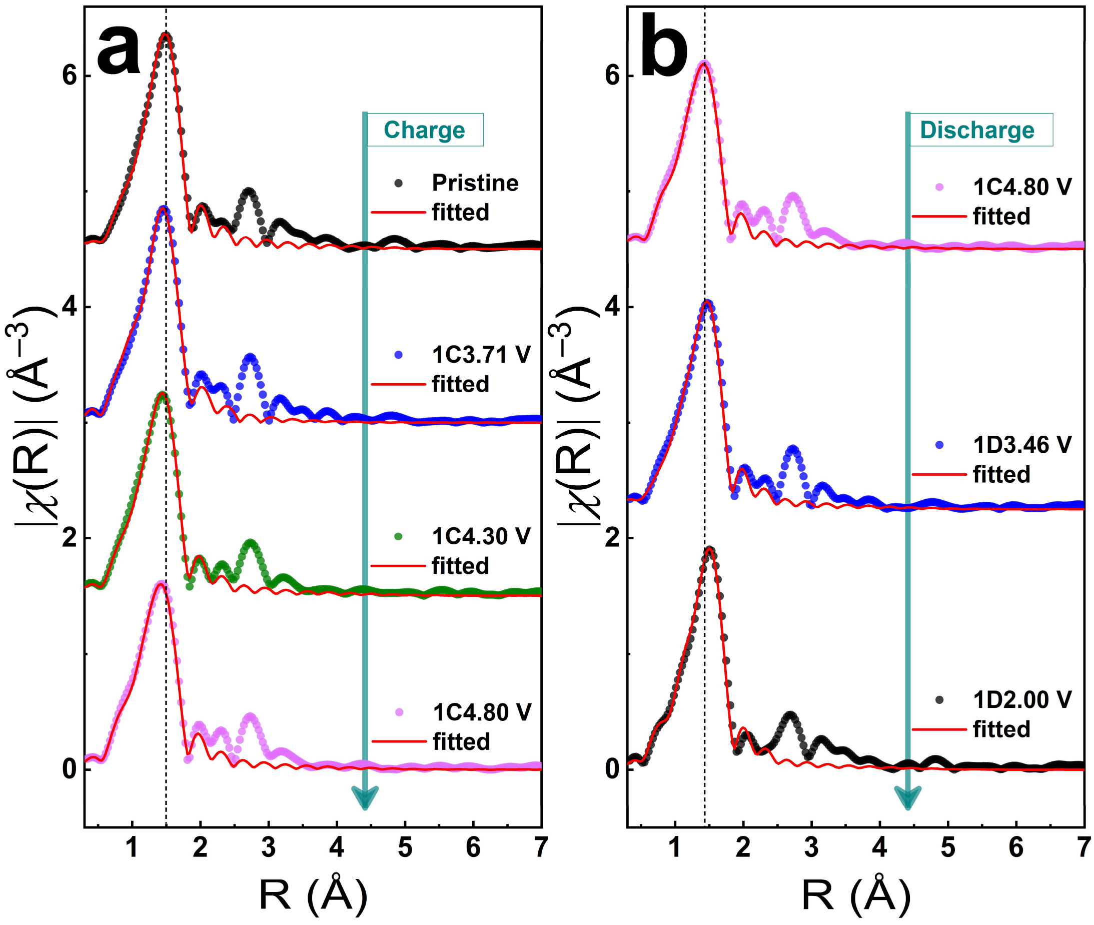

Supplementary Figure 22. The Fourier transform of EXAFS of Ru K-edge. a,b Fitting results for the magnitude of the Fourier transforms performed on k^2-weighted EXAFS oscillations of ID-Li_2RuO_3 during the first charge (a) and discharge (b) processes in the R-range of 1–2 Å (first peak only). E_0 was set to −0.7 eV, and the amplitude reduction factor (S_0^2) was fixed at 0.95. 1C, 1D represent the first charge and discharge, respectively. Two group of Ru–O bonds were considered during fitting based on the presence of two crests in the Ru K-edge XANES spectra shown in Figure 5a.

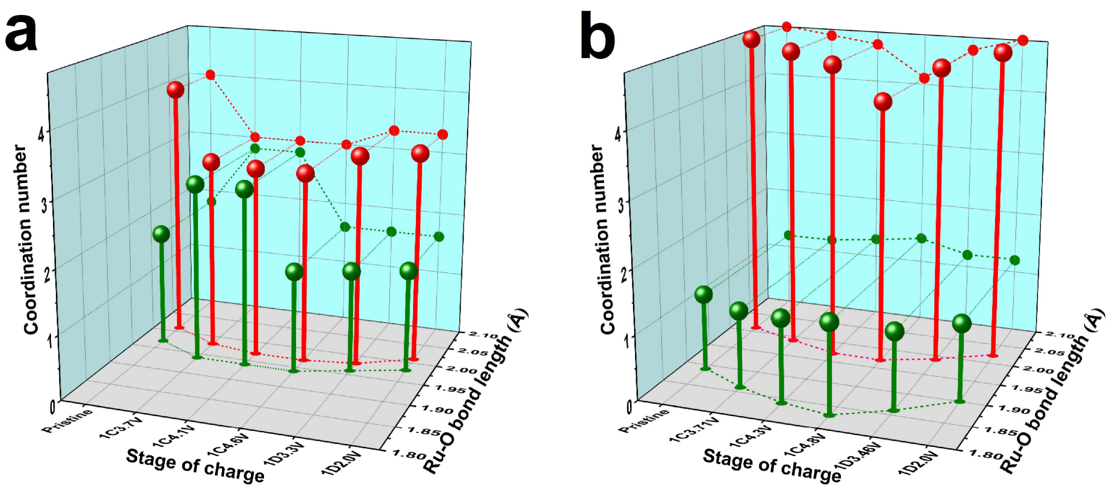

Supplementary Figure 23. The results of Ru–O shell from EXAFS fitting. a,b Fitted Ru–O shell of the Fourier-transformed k^2-weighted EXAFS oscillations of ID-Li_2RuO_3 (**a**), and R-Li_2RuO_3 (**b**). 1C, 1D represent the first charge and discharge, respectively.

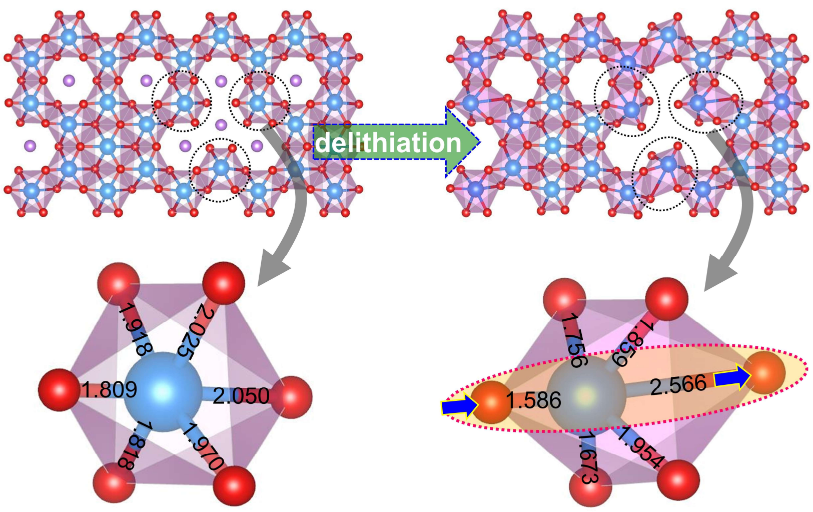

Supplementary Figure 24. Structural response modes of ID-Li_2MnO_3 upon delithiation. Optimized crystal structures and local MnO_6 octahedrons of Li_2MnO_3 and the corresponding delithiated state (Li_0MnO_3) for ID-Li_2MnO_3. The values (in angstrom) on the local structures are the Mn–O bond lengths.

Supplementary Tables

Supplementary Table 1. The total energies (in eV) of the systems with and without oxygen loss at the three kinds of oxygen sites for ID-$Li_{2-x}RuO_3$.

Vacancy sites	$O_{center}[Ru_1Li_5]$	$O_{center}[Ru_2Li_4]$	$O_{center}[Ru_3Li_3]$
$E(Li_2RuO_3–O)$	-532.050	-531.661	-531.660
$E(Li_2RuO_3)$		-539.671	
$E(Li_{1.5}RuO_3–O)$	-492.821	-491.853	-491.639
$E(Li_{1.5}RuO_3)$		-499.557	
$E(Li_1RuO_3–O)$	-453.183	-451.212	-450.621
$E(Li_1RuO_3)$		-458.800	
$E(Li_{0.5}RuO_3–O)$	-405.751	-403.612	-403.285
$E(Li_{0.5}RuO_3)$		-411.281	
$E(Li_{0.25}RuO_3–O)$	-380.719	-379.946	-378.729
$E(Li_{0.25}RuO_3)$		-386.417	
$E(Li_0\ RuO_3–O)$	-355.806	-355.471	-355.043
$E(Li_0RuO_3)$		-360.711	

Supplementary Table 2. The total energies (in eV) of the systems with and without oxygen loss for R-$Li_{2-x}RuO_3$.

Vacancy sites	$O_{center}[Ru_2Li_4]$
$E(Li_2RuO_3–O)$	-533.365
$E(Li_2RuO_3)$	-541.172
$E(Li_{1.5}RuO_3–O)$	-493.482
$E(Li_{1.5}RuO_3)$	-501.158
$E(Li_1RuO_3–O)$	-455.764
$E(Li_1RuO_3)$	-463.509
$E(Li_{0.5}RuO_3–O)$	-406.474
$E(Li_{0.5}RuO_3)$	-411.305
$E(Li_{0.25}RuO_3–O)$	-380.565
$E(Li_{0.25}RuO_3)$	-385.533
$E(Li_0RuO_3–O)$	-355.622
$E(Li_0RuO_3)$	-358.250

Supplementary Table 3. Values used in the calculation of the total energy of the O_2 molecule. The energies of the H_2O and H_2 molecules were obtained from our first-principles study, while TS and ZPE were taken from the literature.[1]

Molecule type	H_2O	H_2	O_2
E (eV)	-14.273	-6.800	-9.426[a]
TS (eV)	0.67	0.41	0.64
ZPE (eV)	0.56	0.27	0.10

[a] The E(O_2) is obtained from Equation S4.

Supplementary Table 4. Crystallographic parameters and structure determination details for ID-Li_2RuO_3 and R-Li_2RuO_3 from XRD and NPD refinement.

	ID-Li_2RuO_3		R-Li_2RuO_3
	XRD refinement	NPD refinement	XRD refinement
Space group	C2/m (No. 12)	C2/m (No. 12)	C2/m (No. 12)
a (Å)	4.9938	5.0065	5.0399
b (Å)	8.6844	8.7082	8.7497
c (Å)	5.1798	5.1848	5.1420
$\alpha = \gamma$ (°)	90.000	90.000	90.000
β (°)	108.587	108.640	109.049
Volume (Å^3)	212.922	214.190	214.330
R_{wp} (%)	5.11	3.58	11.04
R_p (%)	3.88	2.74	8.61
χ^2	4.137	3.897	1.836

Supplementary Table 5. Atomic coordinates of ID-Li_2RuO_3 from XRD refinement.

Atom	Site	Coordinates x	Coordinates y	Coordinates z	Occupation	Uiso ($Å^2$)
Ru(1)	4h	0.000000	0.166728	0.500000	0.701515	0.056554
Li(1)	4h	0.000000	0.166728	0.500000	0.298485	0.029000
Li(2)	2d	-0.500000	0.000000	0.500000	0.403732	0.029000
Ru(2)	2d	-0.500000	0.000000	0.500000	0.596268	0.052420
Li(3)	4g	-0.500000	-0.166720	0.000000	0.999757	0.020071
Ru(3)	4g	-0.500000	-0.166720	0.000000	0.000243	0.055812
Li(4)	2a	0.000000	0.000000	0.000000	0.999784	0.028834
Ru(4)	2a	0.000000	0.000000	0.000000	0.000216	0.056950
O(1)	8j	0.254592	0.168280	0.270732	1.000000	0.023340
O(2)	4i	-0.241671	0.000000	0.272298	1.000000	0.031790

Supplementary Table 6. Atomic coordinates of R-Li_2RuO_3 sample from XRD refinement.

Atom	Site	Coordinates			Occupation	Uiso (Å^2)
		x	y	z		
Ru	4h	0.000000	0.167859	0.500000	1.000000	0.053716
Li(1)	2d	-0.500000	0.000000	0.500000	1.000000	0.019000
Li(2)	4g	-0.500000	-0.158000	0.000000	1.000000	0.021024
Li(3)	2a	0.000000	0.000000	0.000000	1.000000	0.018437
O(1)	8j	0.248934	0.172385	0.267545	1.000000	0.039705
O(2)	4i	-0.233513	0.000000	0.267525	1.000000	0.031569

Supplementary Table 7. Atomic coordinates of ID-Li_2RuO_3 from NPD refinement.

Atom	Site	Coordinates			Occupation	Uiso (Å^2)
		x	y	z		
Ru(1)	4h	0.000000	0.166732	0.500000	0.701204	0.031036
Li(1)	4h	0.000000	0.166732	0.500000	0.298796	0.023590
Li(2)	2d	-0.500000	0.000000	0.500000	0.403153	0.030742
Ru(2)	2d	-0.500000	0.000000	0.500000	0.596847	0.042297
Li(3)	4g	-0.500000	-0.166719	0.000000	0.999751	0.027274
Ru(3)	4g	-0.500000	-0.166719	0.000000	0.000249	0.047763
Li(4)	2a	0.000000	0.000000	0.000000	0.999753	0.027849
Ru(4)	2a	0.000000	0.000000	0.000000	0.000247	0.047832
O(1)	8j	0.254588	0.168285	0.270731	1.000000	0.024047
O(2)	4i	-0.241668	0.000000	0.272302	1.000000	0.028501

Supplementary Table 8. Crystallographic parameters and structure determination details for 4.8V charged ID-Li_2RuO_3 from XRD refinement.

Sample	4.8Vcharged ID-Li_2RuO_3
Space group	C2/m (No. 12)
a (Å)	5.037718
b (Å)	8.718863
c (Å)	4.712646
$\alpha = \gamma$ (°)	90.000
β (°)	90.0097
Volume ($Å^3$)	206.99
R_{wp} (%)	4.22
R_p (%)	3.22
χ^2	4.757

Supplementary Table 9. Atomic coordinates of 4.8 V charged ID-Li_2RuO_3 from XRD refinement.

Atom	Site	Coordinates			Occupation	Uiso (Å^2)
		x	y	z		
Ru(1)	4h	0.000000	0.168110	0.500000	0.701547	0.045805
Li(1)	4h	0.000000	0.168110	0.500000	0.030000	0.039030
Li(2)	2d	-0.500000	0.000000	0.500000	0.030000	0.039030
Ru(2)	2d	-0.500000	0.000000	0.500000	0.596161	0.047284
Li(3)	4g	-0.500000	-0.158000	0.000000	0.100000	0.038628
Ru(3)	4g	-0.500000	-0.158000	0.000000	0.000254	0.045635
Li(4)	2a	0.000000	0.000000	0.000000	0.100000	0.038670
Ru(4)	2a	0.000000	0.000000	0.000000	0.000237	0.048176
O(1)	8j	0.330751	0.170456	0.273423	1.000000	0.039911
O(2)	4i	-0.150312	0.000000	0.276555	1.000000	0.037253

Supplementary Table 10. Detailed fitting results (coordination number (CN), radial distance (R), and Debye–Waller factor (σ^2) for the first shell) for the EXAFS oscillations of R-Li_2RuO_3 during the first charge and discharge processes. E_0 was set to −0.7 eV, and the amplitude reduction factor (S_0^2) was fixed at 0.95. 1C, 1D represent the first charge and discharge, respectively.

State of charge	Pair	CN	R (Å)	σ^2 ($\times 10^{-3}$ Å^2)
Pristine	Ru–O1	1.8	1.97	4.5
	Ru–O2	4.0	2.01	4.2
1C 3.7 V	Ru–O1	2.8	1.94	3.0
	Ru–O2	3.0	1.98	3.4
1C 4.1 V	Ru–O1	2.8	1.94	4.5
	Ru–O2	3.0	1.97	4.5
1C 4.6 V	Ru–O1	1.6	1.94	6.8
	Ru–O2	3.0	1.97	7.0
1D 3.3 V	Ru–O1	1.6	1.96	4.0
	Ru–O2	3.3	1.98	4.3
1D 2.0 V	Ru–O1	1.6	1.98	4.1
	Ru–O2	3.3	2.01	4.3

Supplementary Table 11. Detailed fitting results (coordination number (CN), radial distance (R), and Debye–Waller factor (σ^2) for the first shell) for the EXAFS oscillations of ID-Li_2RuO_3 during the first charge and discharge processes. E_0 was set to −0.7 eV, and the amplitude reduction factor (S_0^2) was fixed at 0.95. 1C, 1D represent the first charge and discharge, respectively.

State of charge	Pair	CN	R (Å)	σ^2 ($\times10^{-3}$ Å^2)
Pristine	Ru–O1	1.2	1.89	2.7
	Ru–O2	4.8	2.01	2.8
1C 3.7 V	Ru–O1	1.2	1.86	2.7
	Ru–O2	4.7	1.99	2.8
1C 4.3 V	Ru–O1	1.3	1.84	2.7
	Ru–O2	4.6	1.97	2.8
1C 4.8 V	Ru–O1	1.4	1.83	2.6
	Ru–O2	4.1	1.97	2.6
1D 3.46 V	Ru–O1	1.2	1.86	2.7
	Ru–O2	4.6	1.99	2.8
1D 2.0 V	Ru–O1	1.2	1.90	2.8
	Ru–O2	4.8	2.02	2.9

Supplementary Notes

Supplementary Note 1. Definition of ΔG for oxygen release. The total energy of the systems with and without oxygen vacancies used in the calculation of oxygen release energy for ID-$Li_{2-x}RuO_3$ and R-$Li_{2-x}RuO_3$ are listed in Supplementary Table 1 and Supplementary Table 2, respectively. The same oxygen vacancy concentration of 1/48 (2% of the total oxygen content) is considered for both ID-$Li_{2-x}RuO_3$ and R-$Li_{2-x}RuO_3$. The Gibbs free energy of oxygen release was calculated as Supplementary Equation 1.

$$\boldsymbol{\Delta G_{(O_2\ release)} = \Delta E_{(O_2\ release)} + \left(-TS_{(O_2)}\right) + ZPE_{(O_2)}} \qquad (1)$$

where $-TS_{(O_2)}$ and $ZPE_{(O_2)}$ of the O_2 gas phase under standard conditions were taken from previous studies.[1,2] and $\boldsymbol{\Delta E_{(O_2\ release)}}$ are defined as Supplementary Equation 2

$$\boldsymbol{\Delta E_{(O_2\ release)} = 2(E_{(X-O)} + \frac{1}{2}E_{(O_2)} - E_{(X)})} \qquad (2)$$

where, $E_{(X-O)}$ and $E_{(X)}$ indicate the calculated total energy of $Li_{2\text{-}x}RuO_3$ with and without oxygen vacancies, respectively, where $E_{(O_2)}$ was modified by the experimental formation energy of water as the binding energy of O_2 molecules from DFT calculations is overestimated.[1,3] The energy of the O_2 molecule was calculated based on Supplementary Equation 3 and 4. Values used in the calculation of $E_{(O_2)}$ were shown in Supplementary Table 3.

$$2H^+ + 2e^- + \frac{1}{2}O_2 \rightarrow H_2O \qquad (\Delta G = -2.46\ eV) \qquad (3)$$

$$\Delta G = \Delta(E - TS + ZPE) = \left[E(H_2O) - E(H_2) - \frac{1}{2}E(O_2)\right] + \left[-TS(H_2O) + TS(H_2) + \frac{1}{2}TS(O_2)\right] + \left[ZPE(H_2O) - ZPE(H_2) - \frac{1}{2}ZPE(O_2)\right] \qquad (4)$$

Supplementary Note 2. Two phase refinement for ID-Li_2RuO_3 sample. In order to evaluate the extent of intralayer disordering, two phase including regular Li_2RuO_3 and ideal intralayer disordered Li_2RuO_3 were used for refinement, which shows that the ratio of regular Li_2RuO_3 and idea intralayer disordered Li_2RuO_3 phases is about 35: 1. The percentage of the idea intralayer disordered Li_2RuO_3 phase is 97.1%, confirming that the ID-Li_2RuO_3 sample is almost the ideal intralayer disordered Li_2RuO_3 phase.

Supplementary References

1 Nørskov, J. K. *et al.* Origin of the overpotential for oxygen reduction at a fuel-cell cathode. *J. Phys. Chem. B* **108**, 17886–17892 (2004).

2 Atkins, P. Atkins' Physical Chemistry, 8th ed. *Oxford University Press: Oxford, U. K.*, 999 (2006).

3 Xiao, R., Li, H. & Chen, L. Density functional investigation on Li2MnO3. *Chem. Mater.* **24**, 4242–4251 (2012).
